# Distribution of lanthanum carbonate in the gastric mucosa confirmed by electron microscopy with a magnified endoscopy: a case report and literature review

**DOI:** 10.1007/s12328-019-01076-5

**Published:** 2019-12-03

**Authors:** Takahiro Yamada, Tsuneo Oyama, Akihisa Tomori, Akiko Takahashi, Kiyokazu Kametani, Satoshi Shiozawa, Hiroyoshi Ota, Tadakazu Shimoda

**Affiliations:** 1 Department of Gastroenterology, Saku Central Hospital Advanced Care Center, 3400-28 Nakagomi, Saku, Nagano 385-0051 Japan; 2 Department of Endoscopy, Saku Central Hospital Advanced Care Center, Saku, Nagano Japan; 3 Division of Instrumental Analysis, Research Center for Human and Environmental Sciences, Shinshu University, Matsumoto, Nagano Japan; 4 Clinical Pathology, Saku Central Hospital Advanced Care Center, Saku, Nagano Japan; 5 Department of Biomedical Laboratory Sciences, School of Health Sciences, Shinshu University School of Medicine, Matsumoto, Nagano Japan; 6 Department of Pathology, Shizuoka Cancer Center, Shizuoka, Japan

**Keywords:** Gastric mucosa, Lanthanum, Retrospective

## Abstract

We describe the case of a 70-year-old man with diabetic nephropathy undergoing hemodialysis. Four years following hemodialysis, he started taking lanthanum carbonate 1500 mg/day and lansoprazole 30 mg/day. Nine years following hemodialysis, he underwent screening esophagogastroduodenoscopy, which demonstrated the presence of the whitish cobblestone-like mucosa in the gastric corpus and multiple reddish depressed lesions with annular whitish mucosa in the antrum. With magnified narrow-band imaging endoscopy, a yellowish–white substance was observed in the villous structure, and subepithelial vessels were observed on the yellowish–white substance. Biopsies were taken from the whitish cobblestone-like mucosa of the upper corpus, a reddish depressed part of the antrum. Histologically, aggregates of cells containing amphophilic fine granular material were found in the mucosal interstitium. These cells stained positive for CD68 and were identified as histiocytes. Since he had been taking lanthanum carbonate for 5 years, we considered the possibility of histiocyte-mediated phagocytosis of lanthanum. Digital mapping via scanning electron microscopy with energy-dispersive X-ray spectrometry showed the presence of lanthanum and phosphorus in the interstitium and cytoplasm of histiocytes. The white, rough mucosa in the gastric body appeared 6 months following the commencement of lanthanum administration and still exists 3 years and 5 months after discontinuation of lanthanum.

## Introduction

Lanthanum carbonate is a powerful phosphate-binding agent used to treat hyperphosphatemia in patients with chronic renal failure. It binds with phosphate groups to form a highly insoluble substance. Lanthanum carbonate itself is insoluble, and its rate of absorption via the intestine is reported to be only 0.00127% [1]. Histopathologically, lanthanum deposition in the gastric mucosa was first demonstrated by Makino et al. in 2015 [2]. To date, numerous case reports have been published on lanthanum deposition [3–17]. Previous reports of endoscopic findings of lanthanum deposition in the gastric mucosa have varied widely. Specifically, Iwamuro et al. reported that gastric mucosa with annular whitish mucosa, diffuse whitish mucosa, and whitish spots visible by endoscopy may be characteristic clinical features of lanthanum deposition [14, 16, 17]. However, no studies have been conducted to address the endoscopic findings of lanthanum deposition in the gastric mucosa using magnified narrow-band imaging (NBI) endoscopy in detail. Furthermore, although previous reports demonstrated the existence of lanthanum by scanning electron microscopy (SEM), they did not describe where lanthanum exists.

## Case report

The patient was a 70-year-old man with no particular complaints. He had a previous history of diabetic nephropathy, cerebral infarction, and colorectal cancer. Four years following hemodialysis, he began taking lanthanum carbonate 1500 mg/day and lansoprazole 30 mg/day. Blood test results revealed severe renal impairment during dialysis treatment, and the patient exhibited elevated levels of magnesium, phosphate, and glycated hemoglobin A1c. Serum *Helicobacter pylori* antibody levels were found to be negative (8.7 IU/ml) (Table 1).

**Table 1:** Laboratory data

Hematology test results	
WBC count 5.6 × 10^3^/μL	UN 56 mg/dL
RBC count 340 × 10^4^/μL	CRE 14.2 mg/dL
Hb level 10.7 g/dL	Na 140 mmol/L
PLT count 12.0 × 10^4^/μL	K 5.6 mmol/L
TP level 6.8 g/dL	Cl 107 mmol/L
ALB level 3.4 g/dL	Ca 9.1 mg/dL
AST level 4 U/L	P 6.6 mg/dL
ALT level 6 U/L	Mg 2.9 mg/dL
ALP level 167 U/L	GLU 149 mg/dL
LDH level 156 U/L	HbA1c 6.6%
Anti-*Helicobacter pylori* immunoglobulin G antibodies 8.7 U/mL	

*WBC* white blood cell, *RBC* red blood cell, *Hb* hemoglobin, *PLT* platelet, *TP* total protein, *ALB* albumin, *AST* aspartate aminotransferase, *ALT* alanine aminotransferase, *ALP* alkaline phosphatase, *LDH* lactate dehydrogenase, *CRE* creatinine, *Na* sodium, *K* potassium, *Cl* chloride, *Ca* calcium, *P* phosphorous, *Mg* magnesium, *GLU* glucose, *HbA1c* glycated hemoglobin A1c

**Fig. 1:**
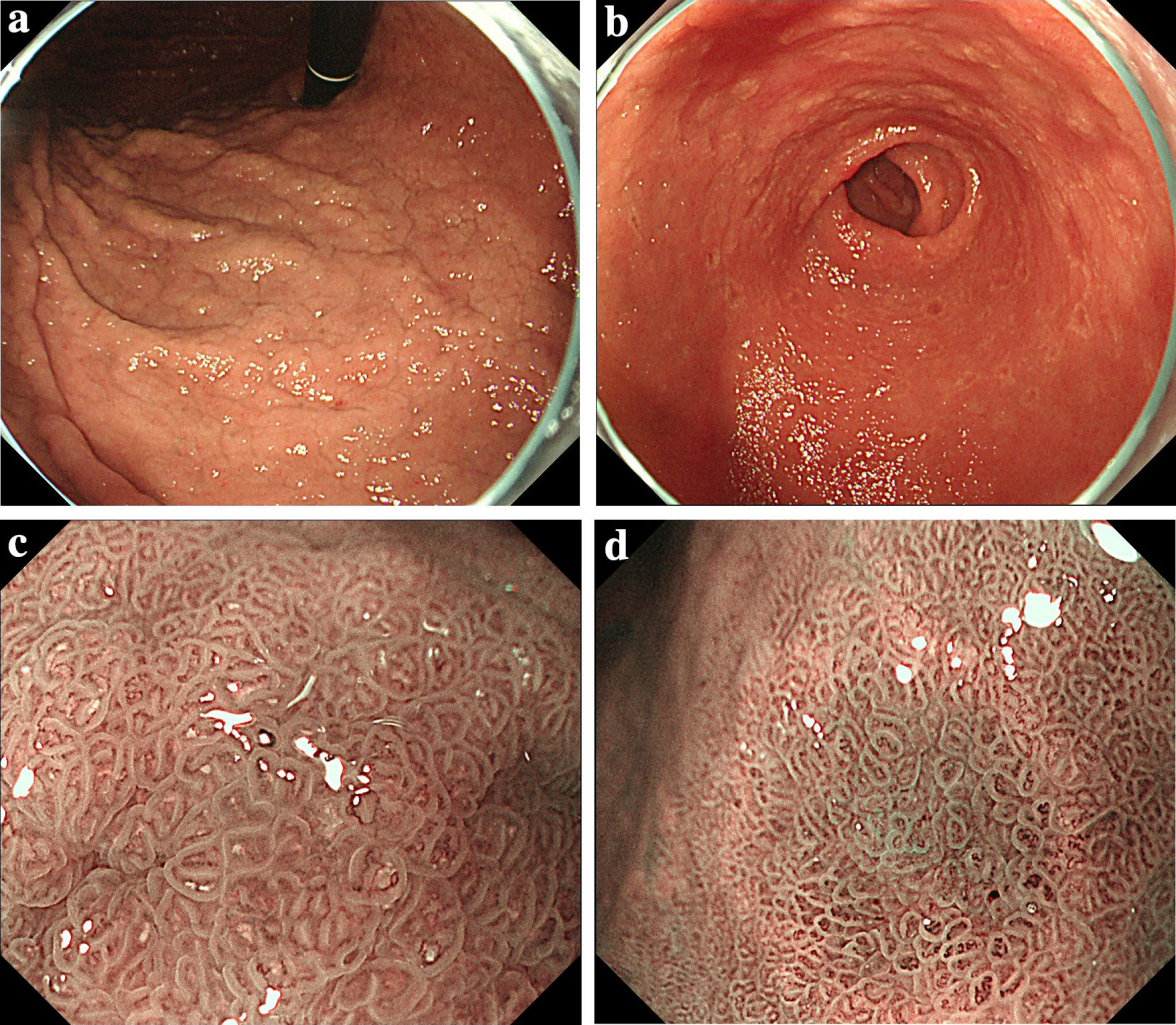
Upper gastrointestinal endoscopic findings. **a** Whitish, rough mucosa is present in the gastric corpus. **b** Depressed red lesions are surrounded by annular yellowish mucosa in the antrum. **c**, **d** With magnified NBI endoscopy, a yellowish–white substance was observed within regular villous-like structures. And a yellowish–white substance was observed above enlarged regular vessels

He underwent screening esophagogastroduodenoscopy (EGD), which revealed whitish cobblestone-like mucosa [18, 19] in the gastric corpus (Fig. 1a) and depressed red lesions surrounded by annular whitish mucosa in the antrum (Fig. 1b). With magnified NBI endoscopy, a yellowish–white substance was observed within regular villous-like structures, and a yellowish–white substance was observed above enlarged regular vessels (Fig. 1c, d).

**Fig. 2:**
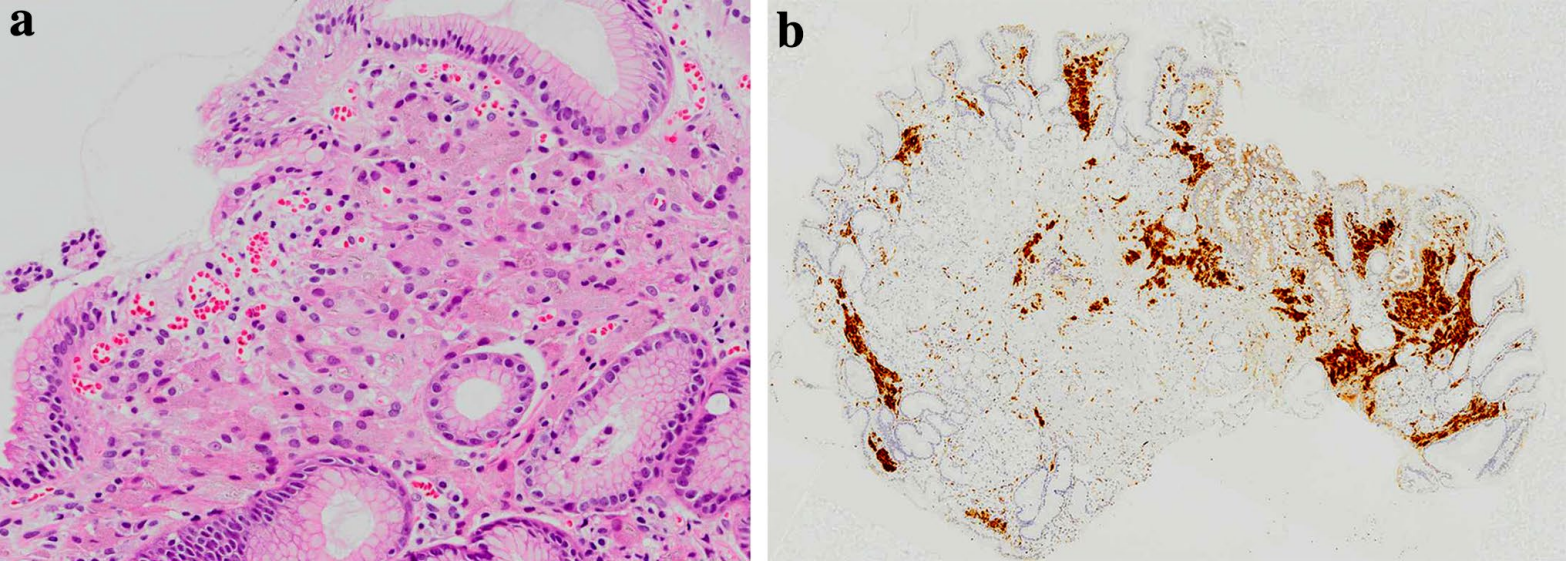
Examination of biopsy tissue specimens. **a**, **b** Aggregates of cells containing amphophilic fine granular material together with coarser brown to deep purple material were observed in the mucosal interstitium of the lamina propria at all biopsy sites by hematoxylin–eosin staining and these cells stained positive for CD68

Biopsies were taken from three locations: an area of whitish, rough granular mucosa on the posterior wall of the upper corpus, a red depressed lesion in the greater curvature of the antrum, and annular whitish mucosa surrounding a depressed lesion. Hyperplasia of parietal cells was observed histologically, which was thought to be due to the lansoprazole ingestion, resulting in the cobblestone-like appearance of the mucosa. And　aggregates of cells containing amphophilic fine granular material together with coarser brown to deep purple material were observed in the mucosal interstitium of the lamina propria at all biopsy sites by hematoxylin–eosin staining (Fig. 2a). These cells stained positive for CD68 and were identified as histiocytes (Fig. 2b). Considering that the patient had been taking lanthanum carbonate, it was hypothesized that the histiocytes might have phagocytosed the heavy metal lanthanum. Thus, we decided to perform SEM–EDS for the element analysis of the deposited materials.

**Fig. 3:**
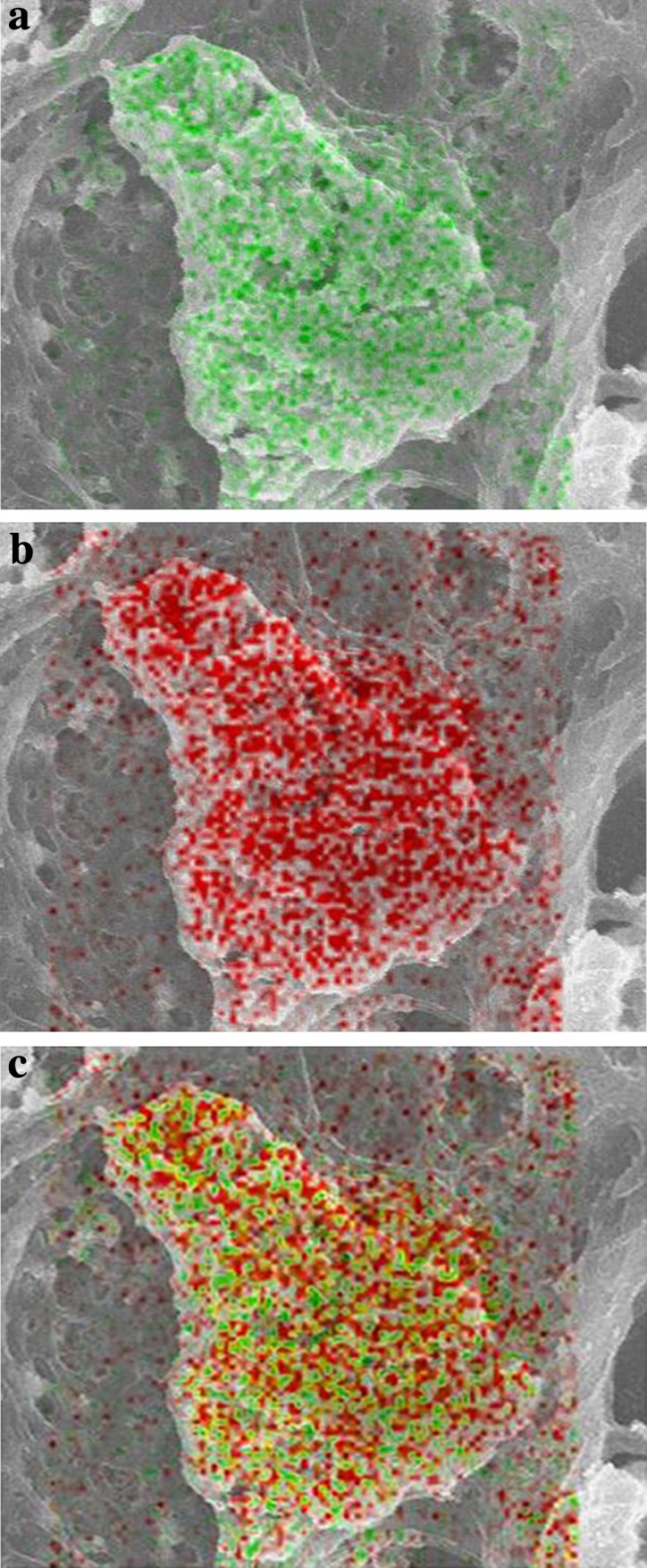
Scanning electron microscopic findings. Green (**a**), red (**b**), and brown spots (**c**) indicate the presence of lanthanum, phosphorus, and a complex of lanthanum and phosphorus, respectively. There is a histiocyte in the center of the figure. Lanthanum, phosphorus, and the complexes are mainly present in histiocytes and partially present in the interstitium

Spectral analysis by EDS characterized the constituent elements of the samples, and deposits of lanthanum and phosphorus were detected. A change in color, observed during the element analysis performed by digital mapping via SEM–EDS, indicated a change in element concentrations. Green and red indicated the presence of lanthanum and phosphorus, respectively, and brown spots formed in the presence of a lanthanum and phosphorus complex. Both lanthanum and phosphorus were primarily found in histiocytes, with partial deposition in the interstitium (Fig. 3a–c).

**Fig. 4:**
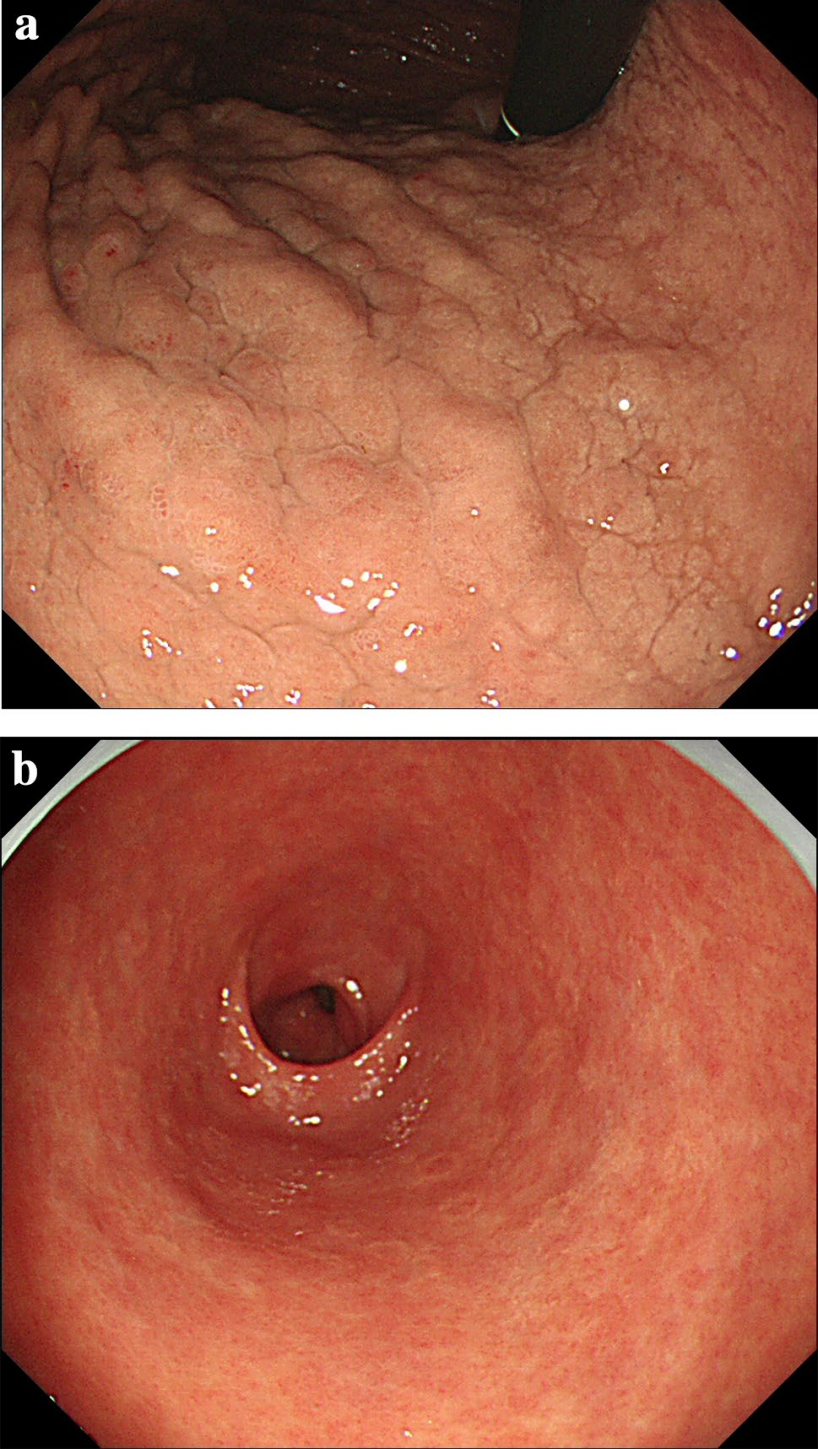
Follow-up upper gastrointestinal endoscopic findings. **a**, **b** Esophagogastroduodenoscopy at 3 years and 5 months after lanthanum discontinuation. The whitish, rough mucosa and depressed lesions improved

**Fig. 5:**
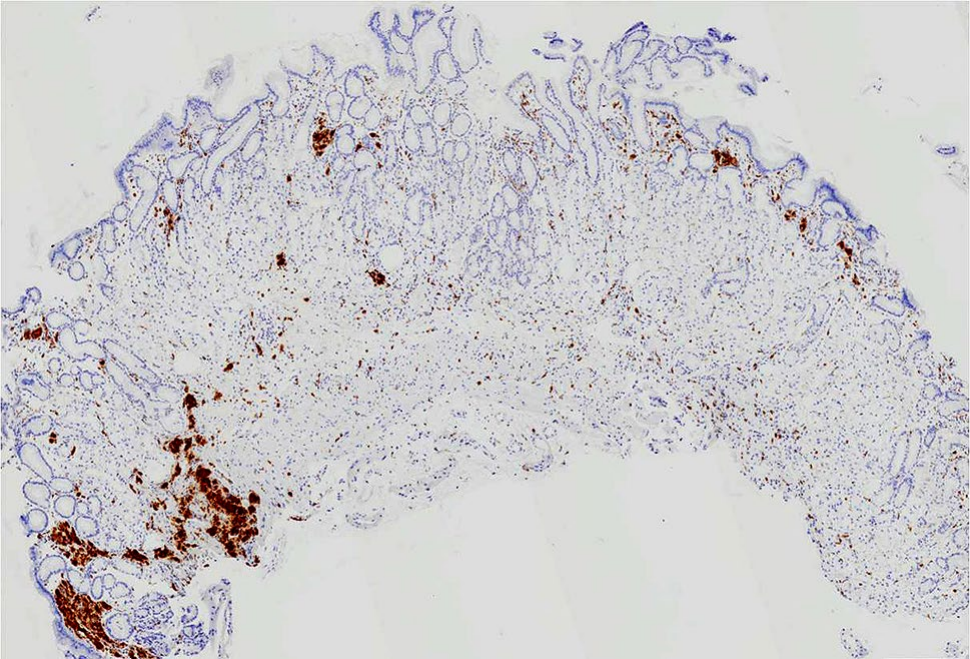
Histology of follow-up biopsy. Number of histiocytes decreased

Subsequently, the patient stopped taking lanthanum, and we continued to perform EGD regularly. Three years and 5 months after discontinuation of lanthanum, whitish rough mucosa and depressed lesions surrounded by annular whitish mucosa improved a little (Fig. 4a, b), and the number of histiocytes has decreased (Fig. 5).

## Discussion

Dialysis patients with chronic kidney disease develop hyperphosphatemia due to their decreased capacity of phosphate excretion. Since hyperphosphatemia causes secondary hyperparathyroidism, it used to be formerly treated with aluminum agents; however, these caused problematic side effects including encephalopathy. In 2009, lanthanum carbonate was approved for coverage by Japanese health insurance. Lanthanum is assumed to be excreted from the body by secretion into bile [1]. It is known that absorbed lanthanum is deposited in the liver and bone, but since the amounts are miniscule, it had been thought not to cause organ damage. However, histopathological deposition of lanthanum has been demonstrated in the gastric mucosa [2].

Some previous reports have described various characteristics, including elevations, erosion, and ulceration [3–10, 12–15], whereas others have described the presence of whitish, rough granular mucosa [2, 11, 14–17]. Table 2 summarizes 51 cases in 13 studies that clearly describe the endoscopic findings of lanthanum deposition in the gastric mucosa, and diagnose it by biopsy. Whitish mucosa was evident in 70.6% of these cases (36/51).

**Table 2:**
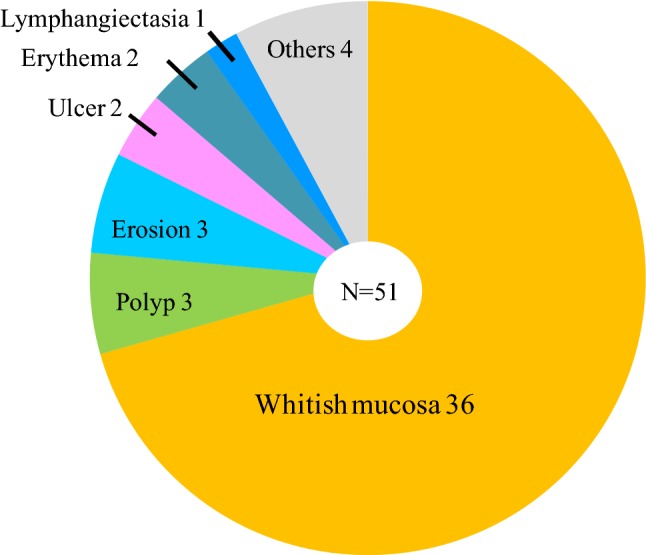
Previously reported endoscopic findings in patients with lanthanum deposition in the gastric mucosa

**Fig. 6:**
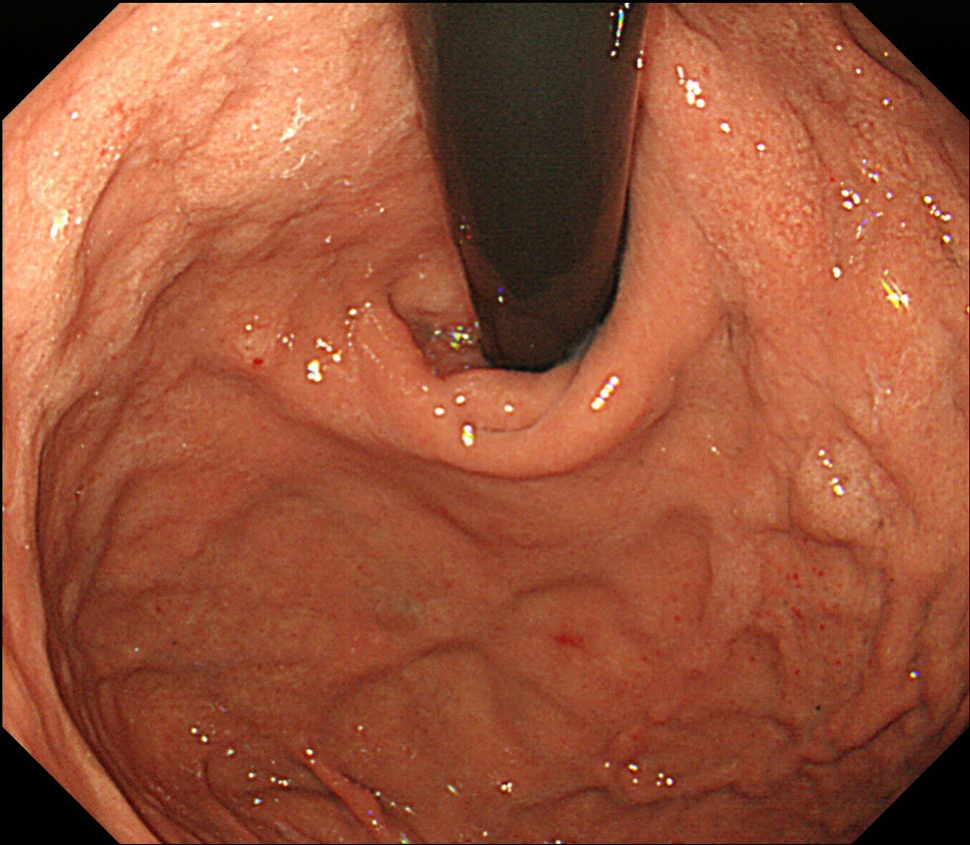
Retrospective review of upper gastrointestinal endoscopy. Esophagogastroduodenoscopy performed 5 years prior to performing the first biopsy shows a whitish cobblestone-like mucosa in the gastric body. Lanthanum was started 6 months prior

The time from the start of lanthanum administration to histological diagnosis varied from 3 to 60 months (mean, 34.5 months). The author suggested that histologically, at least 3 months may be required for the occurrence of lanthanum phagocytosis after the start of lanthanum treatment. In previously reported cases within 12 months starting lanthanum administration with detailed endoscopic findings, whitish mucosa was evident in 83.3% (5/6) of cases in the early stage. This finding suggests that it may be an important indicator of possible lanthanum deposition in the gastric mucosa. Our retrospective review of EGD findings in the present case showed that the whitish cobblestone-like mucosa in the gastric body appeared 6 months after the start of lanthanum administration (Fig. 6).

No studies have been conducted to address the endoscopic findings of lanthanum deposition in the gastric mucosa using magnified NBI endoscopy in detail. In our patient, magnified NBI endoscopy revealed regular villous structures containing a yellowish–white substance; since this was detected below the epithelial vessels, it indicated the presence of a substance in the interstitium of the lamina propria. Based on the result of pathological tests and SEM, we considered that lanthanum had been taken up by histiocytes.

It is important to distinguish between the yellowish–white substance seen by the magnified NBI endoscopy and “white opaque substance” (WOS) [20, 21]. Yao et al. reported WOS obscures the microvessels that are just beneath the epithelium. However, in the present case, we could see microvessels on the yellowish–white substance, meaning that the yellowish–white substance exists beneath the sub-epithelial capillary network. Therefore, it can be concluded that magnified NBI endoscopy can be helpful in distinguishing between the uptake of lanthanum and WOS.

Our patient stopped taking lanthanum carbonate after its deposition was confirmed in the gastric mucosa. However, there have been no significant changes in the endoscopic findings, with the persistence of whitish granular mucosa in the gastric corpus and the depressed lesions surrounded by annular whitish mucosa in the antrum. As needed, we will continue to monitor his condition closely to observe any further changes.

In the present case, lanthanum deposition in the gastric mucosa was demonstrated by SEM–EDS. In SEM, an electron beam is directed at the sample, and electrons and X-rays discharged by the sample are detected to provide a three-dimensional picture of its surface structure. An energy-dispersive X-ray spectroscope is a device attached to a scanning electron microscope that detects characteristic X-rays and obtains information on the elements present in a given sample. “Characteristic X-rays” are multiple X-rays of varying energies characteristic of a particular element, and these are emitted when electrons from an electron beam collide with the atoms in a substance. Since each element emits a different pattern of characteristic X-rays, measuring the energy of the characteristics X-rays enables us to identify the elements present in the area irradiated by the electron beam. There are two methods used for EDS analysis: (1) spectral analysis to determine the elements deposited in the sample, (2) digital mapping for investigating the location of the deposited elements.

In our patient, histiocytes were identified by SEM, and digital mapping by EDS demonstrated the dense deposition of lanthanum phosphate in histiocytes. Lanthanum phosphate was also demonstrated in the interstitium, showing that this agent was not only taken up by histiocytes but also deposited in the interstitium. Previous studies have also demonstrated lanthanum phosphate deposition by SEM, but they did not show lanthanum phosphate deposition in the interstitium. Hence, our results provide evidence suggesting that free lanthanum phosphate in the interstitium is phagocytosed by histiocytes, offering an insight into the process of lanthanum phosphate deposition.

In summary, we reported that the case of lanthanum uptake by histiocytes in gastric mucosa has characteristic findings by magnified NBI endoscopy. We observed a yellowish–white substance within regular villous-like structures and enlarged regular vessels by magnified NBI endoscopy. Additionally, we proved that lanthanum together with phosphorus were densely distributed in histiocytes and sparsely in the interstitium by digital mapping via SEM–EDS.
